# Both cetaceans in the Brazilian Amazon show sustained, profound population declines over two decades

**DOI:** 10.1371/journal.pone.0191304

**Published:** 2018-05-02

**Authors:** Vera M. F. da Silva, Carlos E. C. Freitas, Rodrigo L. Dias, Anthony R. Martin

**Affiliations:** 1 Laboratório de Mamíferos Aquáticos, Coordenação de Biodiversidade, Instituto Nacional de Pesquisas da Amazônia, Manaus, Amazonas, Brazil; 2 Departamento de Ciências Pesqueiras, Faculdade de Ciências Agrárias, Universidade Federal do Amazonas, Manaus, Amazonas, Brazil; 3 Centre for Remote Environments, University of Dundee, Dundee, Scotland, United Kingdom; Sanya Institute of Deep-sea Science and Engineering Chinese Academy of Sciences, CHINA

## Abstract

Obligate river dolphins occur only in the rivers of Asia and South America, where they are increasingly subject to damaging pressures such as habitat degradation, food competition and entanglement in fishing gear as human populations expand. The Amazon basin hosts two, very different, dolphins—the boto or Amazon river dolphin (*Inia geoffrensis*) and the smaller tucuxi (*Sotalia fluviatilis*). Both species have wide geographical ranges and were once considered to be relatively abundant. Their IUCN Red List conservation status of Data Deficient (DD), due to limited information on threats, ecology, population numbers and trends, did not initially cause alarm. However, the development of dolphin hunting to provide fish bait at around the beginning of this millennium broadly coincided with the onset of a widespread perception that numbers of both species were in decline. Consequently, the need for population trend data to inform conservation advice and measures became urgent. This paper presents a 22-year time series of standardised surveys for both dolphins within the Mamirauá Reserve, Amazonas State, Brazil. Analysis of these data show that both species are in steep decline, with their populations halving every 10 years (botos) and 9 years (tucuxis) at current rates. These results are consistent with published, independent information on survival rates of botos in this area, which demonstrated a substantial drop in annual survival, commencing at around the year 2000. Mamirauá is a protected area, and is subject to fewer environmental pressures than elsewhere in the region, so there is no reason to suspect that the decline in dolphins within the Reserve is more pronounced than outside it. If South America's freshwater cetaceans are to avoid following their Asian counterparts on the path to a perilous conservation status, effective conservation measures are required immediately. Enforcement of existing fishery laws would greatly assist in achieving this.

## Introduction

The two very dissimilar cetacean species that occur in the Amazon basin—the Amazon river dolphin, or boto, (*Inia geoffrensis*) and the tucuxi (*Sotalia fluviatilis*)—have a wide geographical distribution and, until recently, were considered abundant in some areas. However, perceptions of a decline in their abundance, combined with increasing evidence of substantial fishery-related mortality and vulnerability to pressures imposed by a rapidly growing human population in the region, have led to fears that these dolphins have been declining in number [1–3]. Both species have long been subject to accidental entanglement in gillnets[3–6]. However, despite being theoretically protected by law in all countries where they occur, botos have additionally been hunted for use as fish bait in Brazil, Colombia, Peru, and Venezuela for 15 years or more [1–3, 7–9]. With a mean inter-birth interval of 4.6 years, and always a litter of a single calf [10], these two species have very limited capacity to compensate for human-related mortality.

Until recent decades, the boto was sheltered from harm to some extent by legends and superstitions, and often released by fishermen when found still alive in their nets [11]. However, some fishermen deliberately killed entangled dolphins, not only because of perceived competition for fish, but also because of damage caused to fishing gear [3, 5, 9, 12]. The relatively new, directed, hunt for botos grew with the use of flesh and blubber as bait for the scavenging catfish *Calophysus macropterus*, known in Brazil as piracatinga, urubu-d’agua and douradinha, and in other Amazonian countries as zamurito, mota and mapurito [12]. This catfish has become widely available commercially, mostly to replace an overfished species in the Colombian market [1, 13]. Much of the Brazilian catch has been exported to that country, but piracatinga is now also sold in cities in Brazil [2, 9, 13].

These dolphins are currently classified by the IUCN Red List as “Data Deficient”, due to the limited amount of current information available on threats, ecology, and population numbers and trends [14, 15]. If perceptions of population declines reflect reality, however, the inability to assign a formal, internationally recognised conservation status to the Amazon's two cetaceans could be masking a significant problem and delaying action to address it.

**Fig 1:**
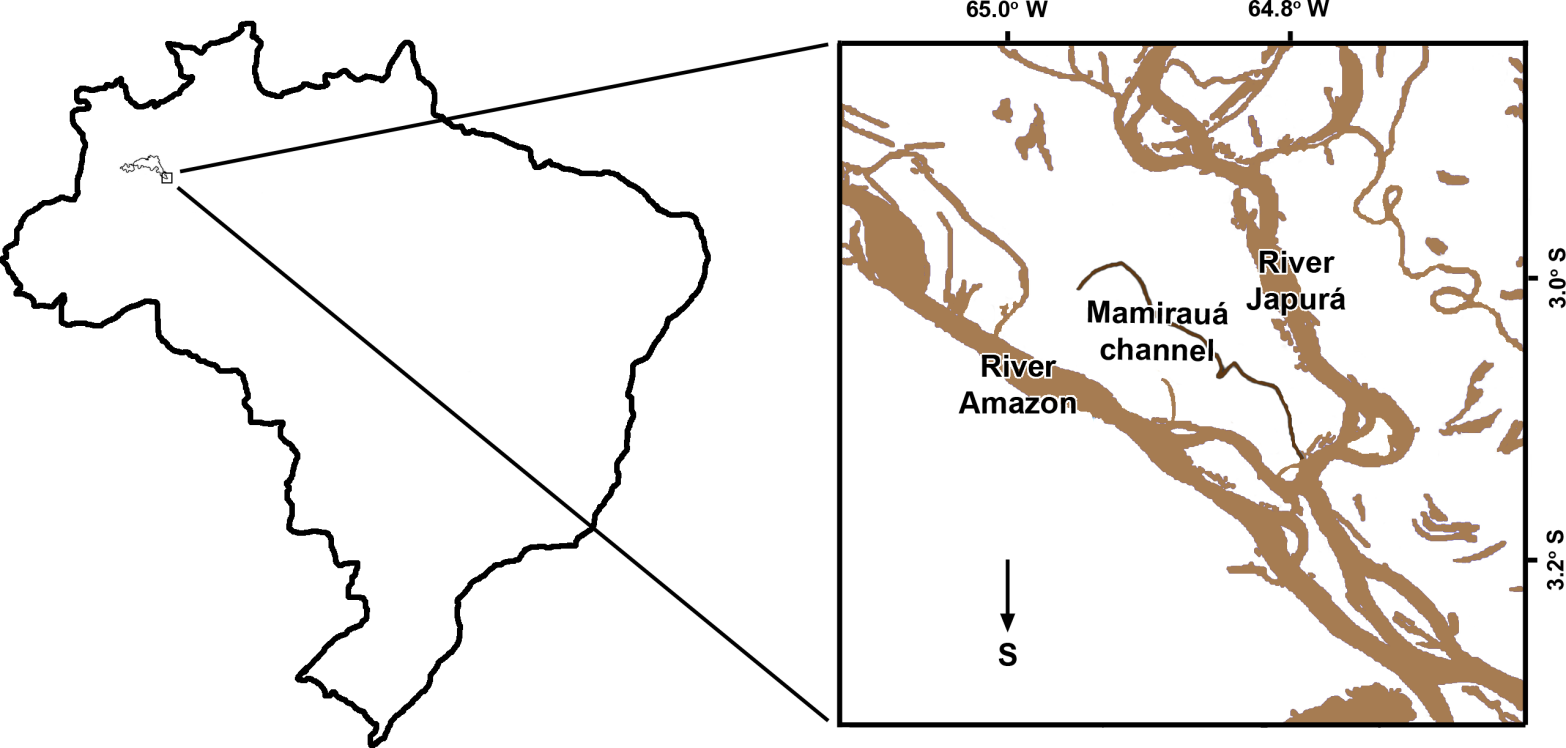
Map of Brazil, showing the location of the study site and (inset) the Mamirauá channel itself.

This paper presents an index of abundance of botos and tucuxis from standardized surveys over a period of 22 yr in the Mamirauá Sustainable Development Reserve (MSDR), one of the largest conservation units in the Brazilian Amazon. Mamirauá is situated in the western Brazilian Amazon, some 500 km west of Manaus (Fig 1). Boto hunting for use as bait is widespread in this region, and gillnets are ubiquitous here, as in the whole of the Amazon basin [3–5, 8, 9, 16].

## Materials and methods

The research on which this paper is based was carried out under permits from the Brazilian Government (IBAMA -Instituto Brasileiro do Meio Ambiente e dos Recursos Naturais Renováveis until 2006–2344/9611-AC; 3552/9312-AC and 2002001.002344/96-11) and from SISBIO (Sistema de Autorização e Informação em Biodiversidade), ICmBio/MMA # 13462–1 to 5; #13157–1 to 4) and the Ethical Committee of the Instituto Nacional de Pesquisas da Amazônia (CEUA/ INPA # 025/2014).

The study site is some 50km by river from the city of Tefé, at 3°20’S; 64°54’W. The MSDR is an area of some 11,000km^2^ between the River Amazon (called the Solimões in this region) and the River Japurá (Fig 1). The reserve comprises V*árzea* habitat and is intersected by lakes and channels. V*árzea* is seasonally flooded lowland forest with a water level range of 10–16m dominating the entire ecology. During the low water season there is much dry land, but rising floodwaters gradually inundate the forest until, at high water, the whole reserve is submerged to a depth of several meters [17–19]. Waterways here comprise some 45km of channels and lakes known as the Mamirauá Lake System. Mamirauá Lake is the largest water body, some 10km long and with an average width of 400m. The Lake is connected to the Japurá River by a channel some 20km long and with an average width of 100m. The surveys discussed in this paper ran along this path, from the mouth of the channel at the junction with the Japurá River to the end of Mamirauá Lake, a distance of *c*. 30 km.

Monthly standardized dolphin surveys were conducted between 1994 and 2017 using a “minimum count” protocol [17]. Each survey consisted of driving a 4m-long aluminum boat powered by a 2-stroke 15 H.P. engine at almost constant speed (ca. 10 km/h), slowing only at sharp curves in the waterway. Boat speed was sufficiently slow as to allow the entire water surface to be visible long enough for any animal present to blow at least once, but not so slow as to allow dolphins to overtake the boat and thus be counted twice.

The survey protocol remained constant throughout the duration of the study, with the same boat and engine type throughout and the same driver from 1999 onwards. Water level affects the abundance of animals present, since they move to the main rivers in the low water season and many return to *Várzea* during the months of high water levels. In *Varzea*, tucuxis remain in channels and lakes, whereas botos often venture into dense flooded forest for short periods [20].

Surveys were always carried out at the same time of day (first thing in the morning), always in the same direction and only in conditions of good visibility (calm water and no rain). Normally three experienced observers (range 2–5) searched for dolphins with the aid of 7x binoculars–one looking only forward, one only backward and a third recording. The scatter plot of number of observers present during a survey on the number of botos and tucuxis counted (S1 Fig) indicated no relationship, and this was confirmed by Pearson correlation coefficients of -0.0329 and -0.142, respectively. Consequently only Time (defined as the number of days since the first survey) and Water Level were included as explanatory variables in the models.

### Model

A generalized linear mixed model was used to investigate changes in the number of encountered botos and of tucuxis, separately, over time between 1994 and 2017. In each case, the natural logarithm of the number of counted dolphins was the response variable. The number of days (Time) since the beginning of the sampling period was the fixed explanatory variable and Water Level was included as a random second-order polynomial function to represent the monomodal hydrological cycle of the Solimões River [21]. Outliers were excluded using the Bonferroni test for outlier detection [22] and model assumptions were judged by visual inspection of standardised residuals (S2–S13 Figs) [21].

The same model was then used with two sub-datasets: the first using data before 2000 (1994 to 1999), corresponding to the years prior to the onset of boto hunting in the region, and the second with data from 2000 onwards—the period of hunting. The same procedure was repeated for tucuxis. A t-test was used to test if the coefficient of the variable Time was equal between these two time intervals.

Models and statistical tests were run using R Statistical Software (R Core Team 2013), employing the CAR [23] and MASS [24] packages for generalised linear models and the APE package [25] to estimate confidence intervals.

## Results

**Fig 2:**
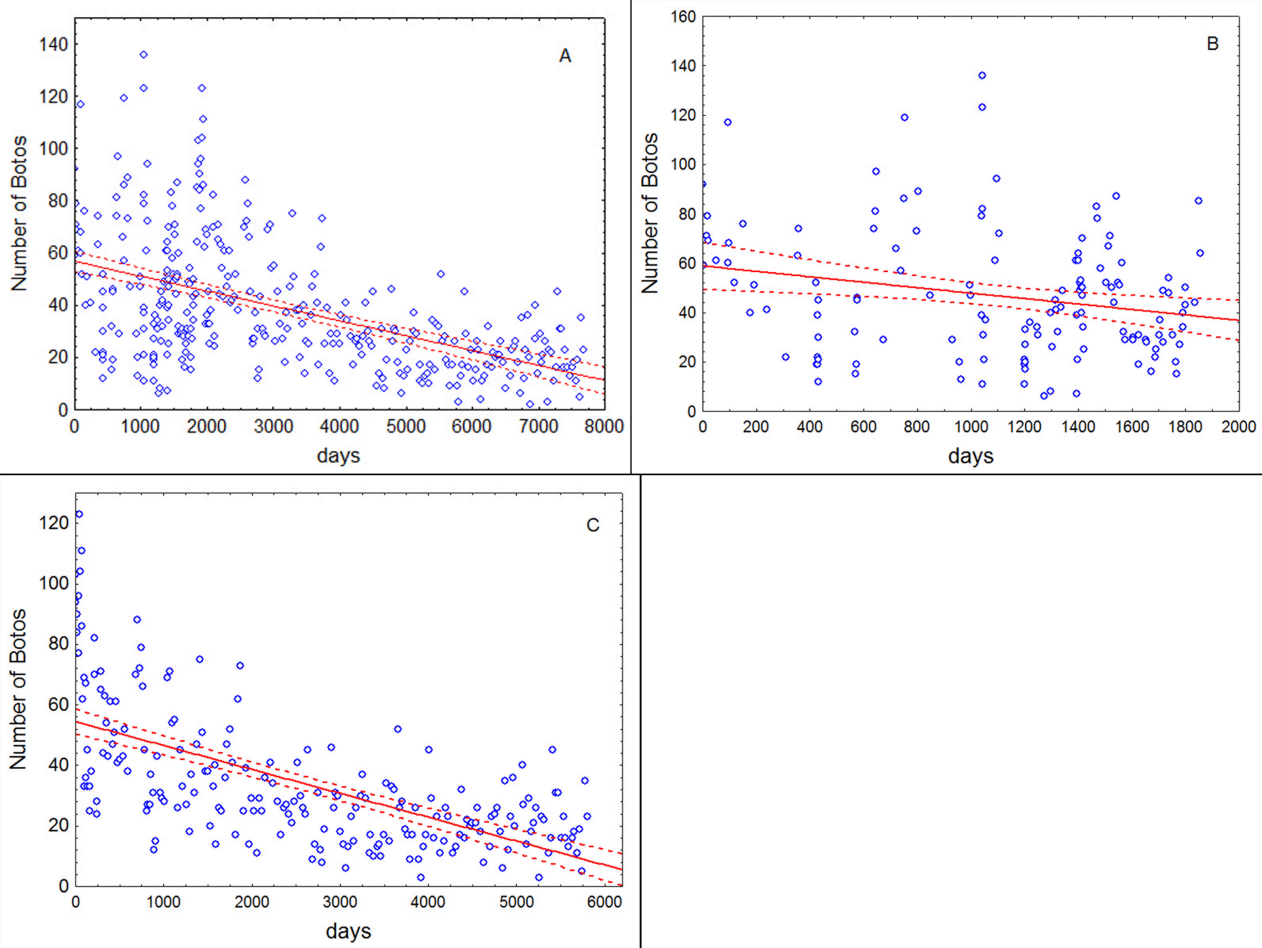
Scatterplots of the number of observed botos per survey as a function of time, including the trend line and 95% confidence intervals estimated by the model. (A) Entire study (Nov 1994—Jan 2017); (B) Nov 1994—Dec 1999; (C) Jan 2000—Jan 2017.

**Fig 3:**
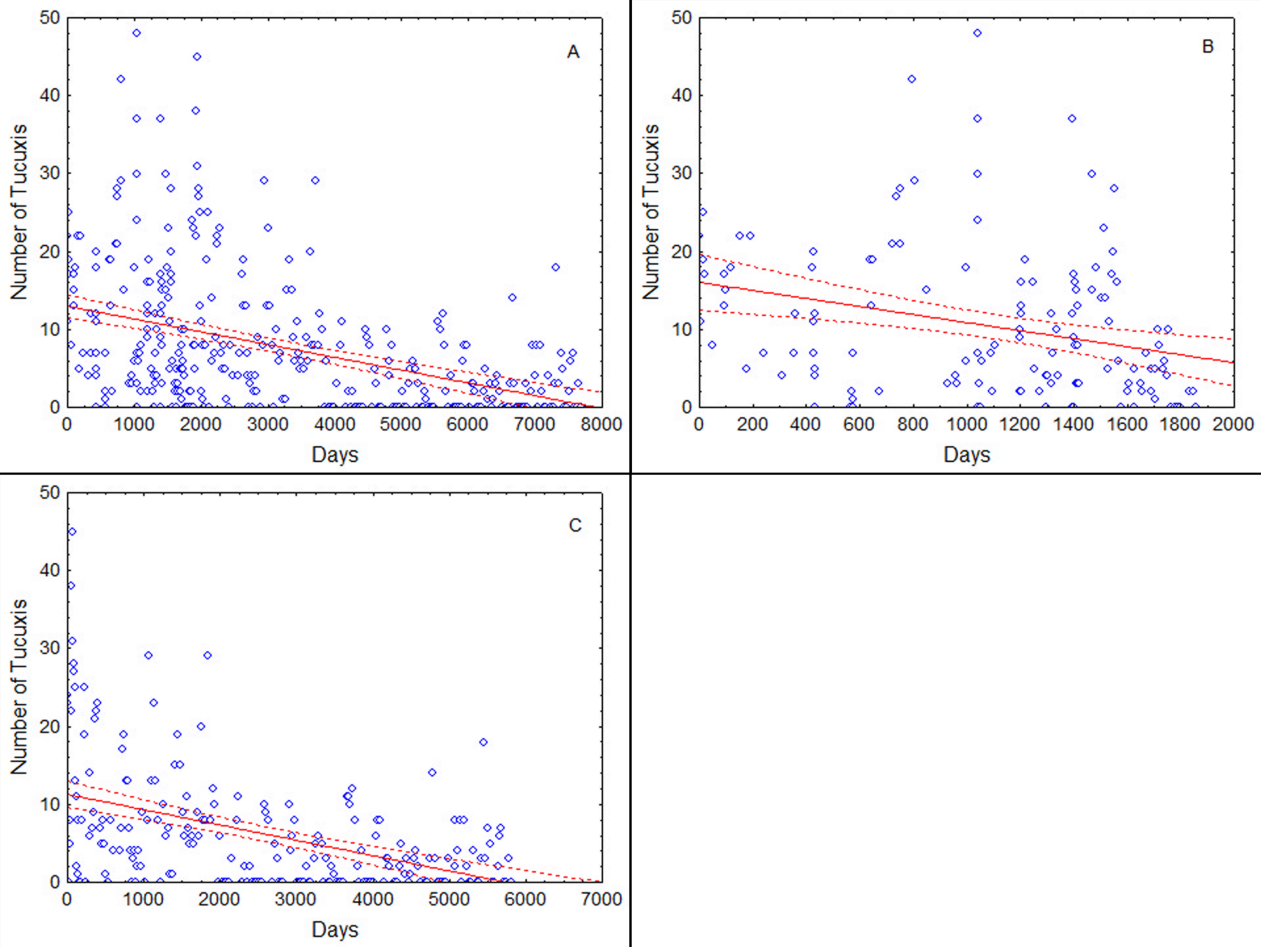
Scatterplots of the number of observed tucuxis per survey as a function of time, including the trend line and 95% confidence intervals estimated by the model. (A) Entire study (Nov 1994—Jan 2017); (B) Nov 1994—Dec 1999; (C) Jan 2000—Jan 2017.

The analysis included 363 sampling events (surveys) between November 1994 and January 2017, with a range of between zero and 136 botos (Fig 2), and between zero and 48 tucuxis (Fig 3). In this time the water level varied between 22.42 m and 38.49 m above mean sea level.

### Botos

The model gave a very good fit to the data over all three time periods (Table 1, Fig 2) and no violation of model assumptions was detected (S2–S7 Figs). In each case a significant linear and quadratic relationship was shown between the number of botos counted and water level. A substantial decline in the number of botos was apparent over the entire sampling period, but closer examination demonstrated that this decline was not statistically significant before the year 2000. From 2000 onwards (Fig 2C), the count diminished at an average daily rate of 0.019% (C.I. 0.016–0.022%), equivalent to 6.7% per year. At this rate, the number of botos counted during the surveys halved every 10.0 years. The coefficient for rate of change of the number of counted botos differed significantly between the pre-hunt (1994–1999) and post-hunt (2000–2017) periods.

**Table 1:** Model results for boto counts. The unit of time is one day, so a coefficient of -0.00015 represents a decline in the number of encountered botos of 0.015% per day, or 5.48% per year during the entire sampling period.

	Nov 1994—Jan 2017		Nov 1994—Dec 1999		Jan 2000—Jan 2017	
	Estimate	p-value	Estimate	p-value	Estimate	p-value
Time	-0.00015	<0.001	-0.00005	0.542 N.S.	-0.00019	<0.001
(95% confidence interval)	(-0.00013, -0.00017)		(-0.00021, +0.00011)		(-0.00016, -0.00022)	
Water Level	0.2991	<0.001	0.299	<0.001	0.3001	<0.001
Water Level^2^	-0.00562	<0.001	-0.0056	<0.001	-0.0055	<0.001
Explained deviance (pseudo R^2^)	97.89%		98.2%		98.29%	
Null deviance (d.f.)	4497.257 (359)		1917.75 (135)		2583.255 (221)	
Residual deviance (d.f.)	94.681 (356)		33.563 (132)		44.191 (218)	
No. surveys / days	361 / 8,083		138 / 1856		221 / 6,206	
Outliers excluded	2		2		3	

### Tucuxis

The number of tucuxis encountered during the surveys diminished significantly with time in all three time periods examined (Table 2, Fig 3). In contrast to botos, however, tucuxi density diminished significantly both before and after the year 2000. The model indicated a reduction in the number of tucuxis over the entire sampling period of 0.021% per day, or 7.4% per year (Fig 3A). This represents a halving of the number counted every 9.04 years. Standard residuals of the model runs are given in the Supplementary Information (S8–S13 Figs). A t-test comparing the coefficients of the variable Time (number of days) rejected the hypothesis that the rate of reduction in the number of counted tucuxis was the same in the periods before 2000 and after 2000 (t = 31.275, d.f. = 354, p < 0.001).

**Table 2:** Model results for tucuxi counts. The unit of time is one day, so a coefficient of -0.00021 represents a decline in the number of encountered tucuxis of 0.021% per day, or 7.67% per year during the entire sampling period.

	Nov 1994—Jan 2017		Nov 1994—Dec 1999		Jan 2000—Jan 2017	
	Estimate	p-value	Estimate	p-value	Estimate	p-value
Time	-0.00021	<0.001	-0.00059	<0.001	-0.00023	<0.001
(95% confidence interval)	(-0.00017, -0.00026)		(-0.00027, -0.00081)		(-0.00016, -0.00030)	
Water Level	0.17057	<0.001	0.1985	<0.001	0.1466	<0.001
Water Level^2^	-0.00306	<0.001	-0.0036	<0.001	-0.0026	<0.001
Explained deviance (pseudo R^2^)	73.43%		81.84%		67.21%	
Null deviance (d.f.)	1392.38 (359)		689.01 (135)		703.36 (221)	
Residual deviance (d.f.)	369.87 (356)		125.06 (132)		230.6 (218)	
No. surveys / days	363 / 8,083		138 / 1,856		221 / 6,206	
Outliers excluded	3		1		3	

## Discussion

The key output of these analyses is the trend in abundance with time rather than absolute numbers; the fact that botos were usually more abundant than tucuxis during these surveys is not important. The tucuxi is more typically a species of the main rivers, whereas lake systems like Mamirauá are a fundamental part of boto ecology [20], so it is likely that a greater proportion of the boto population is within this lake system at any time. Animals of both species enter and leave the Mamirauá system at will, and may range over vast areas of the Amazon; none remain inside the system perpetually. Dolphins within the study area are but a subsample of the population in the region as a whole.

In both species there were statistically significant profound, sustained reductions in abundance. What can be inferred from these results? The first point to make is that the waterway covered by these surveys has not undergone any substantial change during the two decades of this study. Commercial fishing was banned in the reserve before the study began [19], and the ban was observed throughout the study period with inconsequential exceptions. The number of humans living along the study waterway and using it for subsistence fishing has remained low [26]. Other fish predators, such as egrets, cormorants (authors' pers. obs.), caimans (João Valsecchi, Mamirauá Institute, pers. comm.) and the large air-breathing fish *Arapaima gigas* [27] remain abundant, suggesting that populations of the small and medium sized fish favored by dolphins are healthy. The region as a whole has not been affected by any dams or changes in industrial fishing, logging, mining or shipping. The density of small boats has increased in part of the waterway as the local human population has become wealthier, but that is the case everywhere in the region. In short, there is no obvious reason other than fishery-related mortality for the abundance of dolphins in Mamirauá to decline, and there are certainly no apparent reasons for dolphins to leave the relatively quiet, protected waters of Mamirauá for other lake systems. On the contrary, it would not be surprising if dolphins were moving from other more heavily exploited and disturbed areas to Mamirauá. If so, the decline in the original dolphin populations may be even higher than is evident in these data.

The substantial, sustained reduction in the number of botos shown by the current study is consistent with independent analysis of the annual survival rate of dolphins in this population. Survival rates in the years prior to deliberate hunting were some 7% higher than during the hunt (97% compared to 90%) [13]—a change that would very likely represent the difference between a healthy and declining population. There seems little doubt that the loss of botos is real, and that the most likely cause of the loss is both directed and incidental mortality caused by human fisheries. Since the year 2000, when the directed hunt was known to be underway, our results show that well over half of the boto population of the Mamirauá Reserve and surrounding area has been lost.

As steep as is the decline in botos, that of tucuxis is even greater. Hunting for this species is almost certainly less intense than for botos [8], but tucuxis are smaller, less powerful and less able to escape entanglement in fishing nets. The use of gillnets locally, and in the region as a whole, has increased substantially during the two decades of this study [28]. Human populations in Amazonia are growing rapidly, as is the demand for fish. Almost every house on the margins of rivers has gillnets in evidence. As an example of the hazards faced by cetaceans in the region, and flagrant disregard for the law, the channel through which cetaceans and fish must pass to enter the Mamirauá lake system from the main river is often completely closed off by gillnets throughout much of the night. In these circumstances, cetacean entanglement is inevitable, and all evidence of it would be gone by first light. There is no reason to think that fishery practice in this channel is any more damaging to dolphin populations than elsewhere. If anything, the statutory protected status of Mamirauá, and the fact that many local people earn a living from the existence of the Reserve, may be expected to diminish illegal fishing and hunting in comparison with unprotected areas [16].

Few studies have attempted to quantify the level of fishery-related dolphin mortality in the Amazon, not least because fishers are rarely candid about what they know of the deaths of legally protected species, and carcasses quickly disappear due to human use, scavenging, decomposition, or being washed away by river currents. A study based in the same region as ours, and acknowledged by the authors as grossly under-representing the true number of cetacean deaths [5], demonstrated both that accidental mortality in nets is widespread and that tucuxis are more commonly killed than botos. Given what is known about the ubiquitous, heavy use of fishing nets in this region and the vulnerability of tucuxis to fatal entanglement, the extremely rapid reduction in tucuxi numbers shown in this study is not only plausible but arguably to be expected.

The area over which these results are representative is unknown, simply because no similar data series is available from elsewhere. However, the likely driver of the population declines evident in and around Mamirauá—fishery-related mortality- is known to be present not only throughout the Brazilian Amazon (e.g. Manacapuru, c.550 km by river from Mamirauá, where just two traders in one community handled 300 dolphins per year [3]) but in all countries where these species live [1, 7]. As such, it appears likely that similarly striking losses of dolphins have occurred on a huge geographical scale.

This study represents the first quantified assessment of medium-term population trends of the Amazon's two freshwater dolphins in any part of the basin. The results are profoundly concerning and show rates of decline among the most severe of any measured in a cetacean population since the early years of modern whaling. It is clear that without rapid, effective changes in fishery practices, populations of both the boto and tucuxi will continue their rapid decline, at least in this core part of their range and probably much more widely. Were the IUCN Red List criteria to be applied on the basis of this study, the population of both species would be classified as Critically Endangered (CR), due to 'an observed, estimated, inferred, projected or suspected population size reduction of ≥ 80% over any 10 year or three generation period, whichever is longer, where the time period must include both the past and the future, and where the reduction or its causes may not have ceased' [29]. Here, generation length is assumed to be 13.3 yr in botos and 13.9 yr in tucuxis [30] yielding 3-generation declines of 94% in botos and 97% in tucuxis at rates of loss pertaining since the year 2000 (Tables 1 & 2). Modeling work by Huang et al. [31] indicated that 'traditional census survey techniques [for freshwater cetaceans] are unlikely to detect early signs of population decline before a critical level is reached', and this does indeed appear to be the case for both the boto and tucuxi.

Dramatic reductions in populations of obligate freshwater cetaceans are not new. Indeed in Asia, the only other part of the world where they occur, declines are the norm, culminating in the extinction of the baiji, or Chinese river dolphin (*Lipotes vexillifer*) [32] and assessment of the Ganges/Indus river dolphin *Platanista gangetica* as Endangered under IUCN Red List criteria [33]. Hitherto, however, South America's freshwater dolphins have been perceived to be relatively abundant, and concern about reported fishery mortality has been relatively muted. The one exception to this is a recent temporary Brazilian ministerial regulation which was intended to put an end to the hunting of dolphins by prohibiting the commercial exploitation of the fish species for which dolphin meat is used as bait. This prohibition[34] came into force at the start of 2015 and is due to be re-evaluated after five years, although there is increasing evidence that it is being widely ignored [35, 36]. Regardless of its current effectiveness, this regulation represents official recognition of the damage being done to the Amazon's dolphins by fisheries, and is an important first step in facilitating recovery. However, without an indefinite extension and strict enforcement of this regulation, combined with observance of existing laws on gillnet use, the dolphins of the Amazon seem very likely to follow the freshwater dolphins of Asia on the path to extinction.

## Supporting information

S1 Fig Plots of dolphins seen during a survey v number of observers: (left) botos, and (right) tucuxis. (DOCX)

S2 Fig Graphical test of model assumptions. Boto entire time series: histogram of standardised residuals.
(DOCX)

S3 Fig Graphical test of model assumptions. Boto entire time series: scatterplot of standardised residuals against predicted values.
(DOCX)

S4 Fig Graphical test of model assumptions. Boto before 2000: histogram of standardised residuals.
(DOCX)

S5 Fig Graphical test of model assumptions. Boto before 2000: scatterplot of standardised residuals against predicted values.
(DOCX)

S6 Fig Graphical test of model assumptions. Boto after 2000: histogram of standardised residuals.
(DOCX)

S7 Fig Graphical test of model assumptions. Boto after 2000: scatterplot of standardised residuals against predicted values.
(DOCX)

S8 Fig Graphical test of model assumptions. Tucuxi entire time series: histogram of standardised residuals.
(DOCX)

S9 Fig Graphical test of model assumptions. Tucuxi entire time series: scatterplot of standardised residuals against predicted values.
(DOCX)

S10 Fig Graphical test of model assumptions. Tucuxi before 2000: histogram of standardised residuals.
(DOCX)

S11 Fig Graphical test of model assumptions. Tucuxi before 2000: scatterplot of standardised residuals against predicted values.
(DOCX)

S12 Fig Graphical test of model assumptions. Tucuxi after 2000: histogram of standardised residuals.
(DOCX)

S13 Fig Graphical test of model assumptions. Tucuxi after 2000: scatterplot of standardised residuals against predicted values.
(DOCX)

S1 Database One line per survey, comprising the following fields: Survey number, Date (dd/mm/yy), Date, boto count, tucuxi count, number of observers, water level (m above sea level). (CSV)
