# Supplementary figures and images for: Both cetaceans in the Brazilian Amazon show sustained, profound population declines over two decades
Source: PLoS One. 2018 May 2;13(5):e0191304. doi: 10.1371/journal.pone.0191304 (PMC5931465; doi:10.1371/journal.pone.0191304)

# Supporting Information.

S1 Fig.


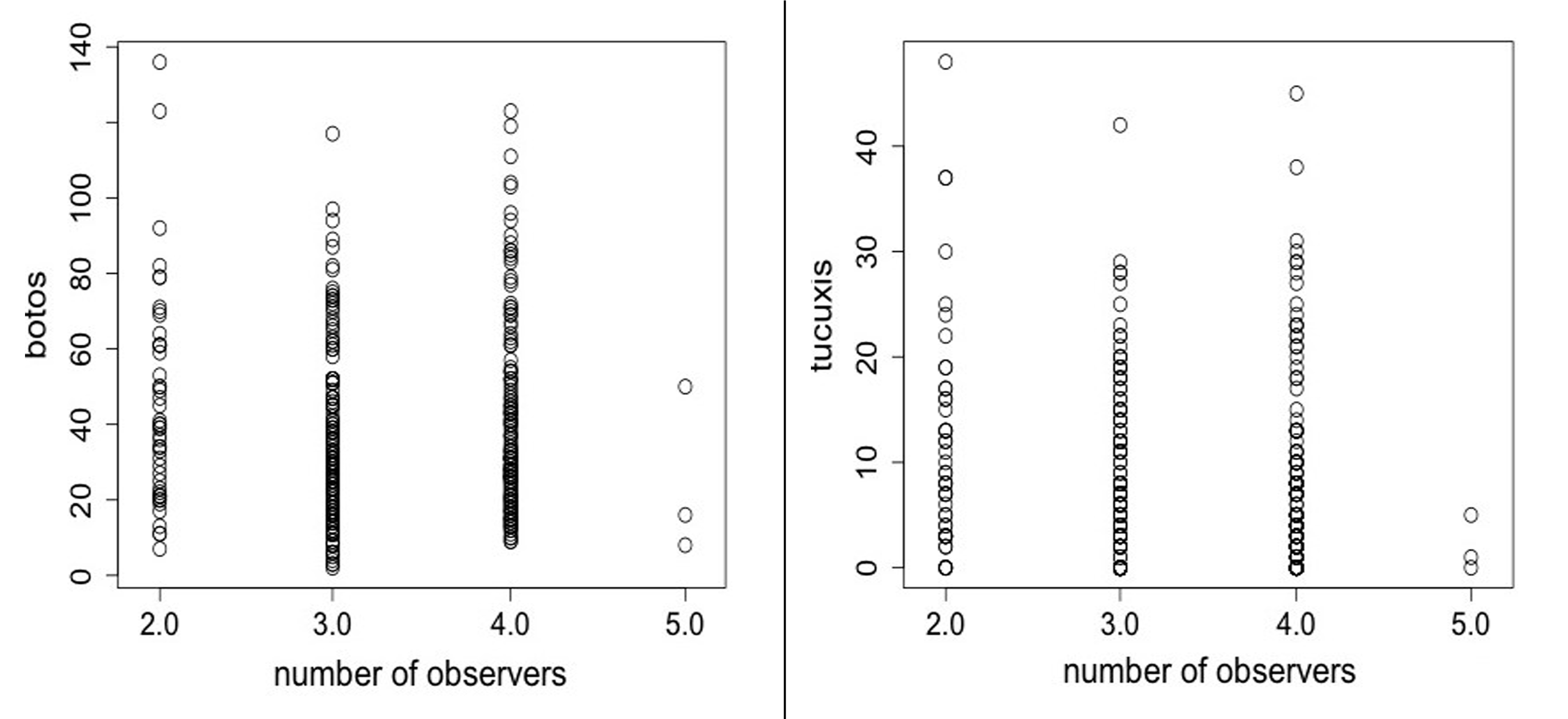

Supplement: S1 Fig — (DOCX) [file pone.0191304.s001.docx]
