# Supplementary material for: Both cetaceans in the Brazilian Amazon show sustained, profound population declines over two decades
Source: PLoS One. 2018 May 2;13(5):e0191304. doi: 10.1371/journal.pone.0191304 (PMC5931465; doi:10.1371/journal.pone.0191304)
Supplement: S9 Fig — Tucuxi entire time series: scatterplot of standardised residuals against predicted values. (DOCX) [file pone.0191304.s009.docx]

S9 Fig.
