# Supplementary material for: Both cetaceans in the Brazilian Amazon show sustained, profound population declines over two decades
Source: PLoS One. 2018 May 2;13(5):e0191304. doi: 10.1371/journal.pone.0191304 (PMC5931465; doi:10.1371/journal.pone.0191304)
Supplement: S12 Fig — Tucuxi after 2000: histogram of standardised residuals. (DOCX) [file pone.0191304.s012.docx]

S12 Fig.
